# Co-evolving adiposity and frailty trajectories shape stroke risk in middle-aged and older adults

**DOI:** 10.1016/j.isci.2026.116954

**Published:** 2026-08-19

**Authors:** Zheng-Jun Wu, Qian-Hui Hu, Ming-Fang He, Qiu-Xia Liang, Jin-Rong Zhang, Hong Liu

**Affiliations:** 1 Department of Cerebrovascular Disease, Guang Yuan Central Hospital, No. 16 Jin Xiang Zi, Li Zhou District, Guangyuan, Sichuan Province 628000, China; 2 Department of Anesthesiology, Guang Yuan Central Hospital, No. 16 Jin Xiang Zi, Li Zhou District, Guangyuan, Sichuan Province 628000, China

**Keywords:** frailty, obesity, stroke, trajectory modelling, cohort study

## Abstract

Obesity and frailty are independently associated with stroke risk, but their joint longitudinal evolution and combined prognostic significance remain unclear. Using harmonized data from four nationally representative aging cohorts (China health and retirement longitudinal study [CHARLS], health and retirement study [HRS], English longitudinal study of aging [ELSA], and survey of health, aging and retirement in Europe [SHARE]), we applied group-based multi-trajectory modeling to 41,717 stroke-free participants aged ≥45 years to identify distinct joint body mass index (BMI)-frailty index trajectories. Five trajectory subgroups were identified in each cohort. Over a median follow-up of 11.0 years, the obesity and high-frailty group consistently demonstrated the highest stroke risk across all cohorts (hazard ratios [HRs], 2.86–7.87), whereas the overweight and low-frailty group showed protective associations (HRs, 0.42–0.60). High frailty was the dominant risk modifier within each BMI category. These findings were robust across extensive sensitivity analyses and indicate that joint BMI-frailty trajectory assessment provides superior stroke risk stratification compared with either measure alone.

## Highlights

• Five joint body mass index and frailty trajectory groups were identified across four cohorts
• Obesity with high frailty showed the highest stroke risk in all cohorts
• Overweight with low frailty was linked to lower stroke risk across cohorts
• High frailty was the dominant stroke risk modifier within each weight category

## Graphical abstract

**Figure:**
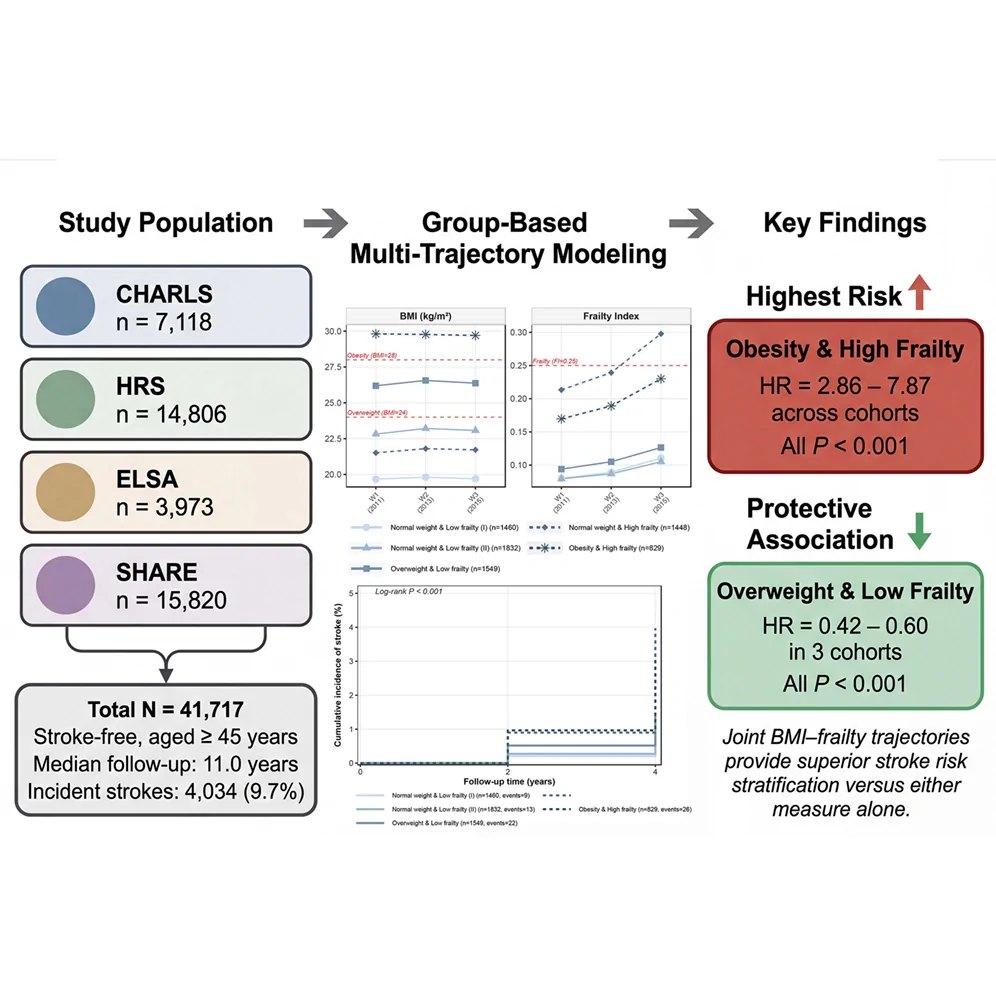

## Teaser

Health sciences

## Introduction

Stroke remains one of the leading causes of death and long-term disability worldwide, imposing a substantial burden on healthcare systems and aging societies.^1^ As global populations age, the prevalence of stroke continues to rise, particularly among older adults who frequently present with multiple comorbidities and diminished physiological reserves.^1^ Among the age-related conditions that complicate stroke risk and recovery, frailty—a state of increased vulnerability to stressors, resulting from cumulative decline across multiple organ systems—has emerged as a critical determinant of adverse cerebrovascular outcomes.^2^ Concurrently, obesity has been recognized as a major modifiable risk factor for stroke, yet its relationship with post-stroke outcomes is complicated by the so-called “obesity paradox,” wherein overweight or mildly obese patients may paradoxically exhibit lower mortality than their normal-weight counterparts.^3^

Accumulating evidence has established that frailty is prevalent in stroke populations, with a pooled pre-stroke frailty prevalence of approximately 24.6%, and that both the presence and severity of frailty are independently associated with increased stroke risk, higher mortality, and poorer functional recovery.^4^^,^^5^ A large observational study of over 253,000 participants demonstrated a dose-response relationship between hospital frailty risk scores and stroke incidence, and Mendelian randomization analyses have further supported a potential causal link between genetically determined frailty and stroke risk.^6^ With respect to the obesity-frailty nexus, meta-analytic evidence indicates that both obesity and underweight status are associated with an elevated risk of frailty in community-dwelling older adults, with obese individuals having a 40% higher risk compared with those of normal weight.^7^ Moreover, the metabolic heterogeneity of obesity plays a pivotal role in frailty progression: metabolically unhealthy overweight or obesity accelerates frailty accumulation, whereas metabolically healthy overweight or obesity does not, suggesting that metabolic status rather than adiposity alone drives this relationship.^8^

Despite these advances, several important knowledge gaps persist. First, most existing studies have examined the associations of obesity and frailty with stroke risk in isolation, without considering their joint longitudinal trajectories.^9^ Second, the temporal dynamics of body mass index (BMI) and frailty over extended follow-up periods and their combined influence on incident stroke remain poorly characterized across diverse populations.^10^ Third, while cross-sectional and short-term longitudinal studies have linked individual frailty or obesity phenotypes to cerebrovascular events, no study has employed group-based multi-trajectory modeling to simultaneously capture the co-evolution of BMI and frailty and evaluate its prognostic significance for stroke across multiple international cohorts.^11^ This gap is particularly important given the known cross-national heterogeneity in BMI distributions, frailty burden, and stroke epidemiology.

To address these limitations, we conducted a multi-cohort longitudinal study using harmonized data from four nationally representative aging surveys—China health and retirement longitudinal study (CHARLS), health and retirement study (HRS), English longitudinal study of aging (ELSA), and survey of health, aging and retirement in Europe (SHARE)—comprising 41,717 participants across 18 countries. We applied group-based multi-trajectory modeling to identify distinct joint BMI-frailty trajectory subgroups and examined their associations with incident stroke using Cox proportional hazards regression with progressive covariate adjustment. We further evaluated the robustness of findings through extensive subgroup and sensitivity analyses, including alternative adiposity and frailty measures and competing risk models.

## Results

### Baseline characteristics of study participants

**Figure 1:**
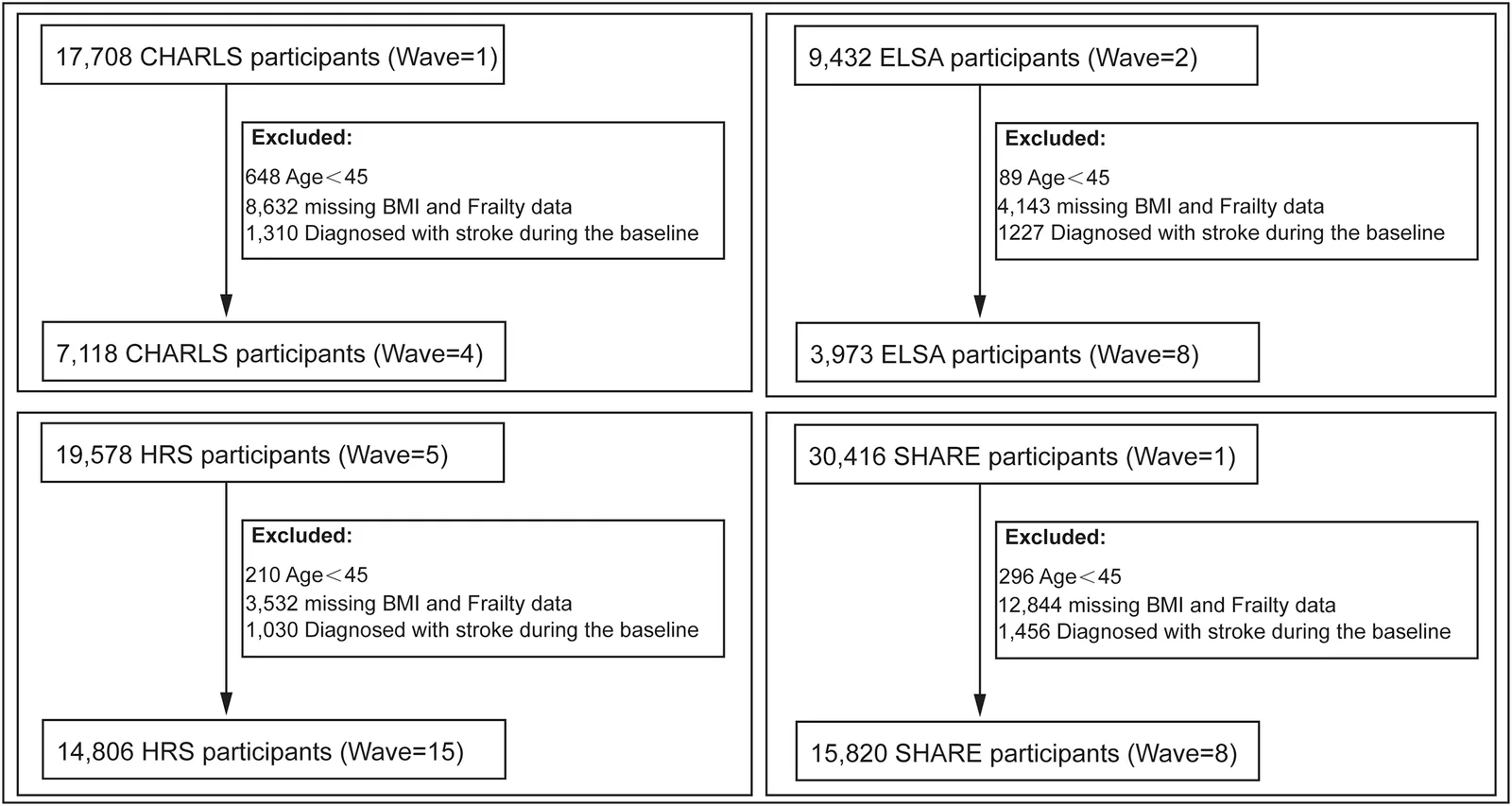
Flowchart of participant selection and exclusion criteria across four cohort studies

After applying the exclusion criteria, 41,717 participants were included in the final analytic sample: 7,118 from CHARLS, 14,806 from HRS, 3,973 from ELSA, and 15,820 from SHARE (Figure 1). The overall median age was 62.00 years (interquartile range [IQR], 56.00–69.00), and 56.7% were female. CHARLS participants were the youngest (median, 59.00 years) with the highest proportion of rural residents (68.4%) and current smokers (30.6%), whereas HRS participants were the oldest (median, 64.00 years) with the highest prevalence of hypertension (42.9%), arthritis (49.5%), and cancer (9.6%). Drinking prevalence varied markedly across cohorts (standardized mean difference [SMD] = 0.679), ranging from 32.2% in CHARLS to 92.2% in ELSA. Depression was reported by 24.5% of participants overall, with the highest rate observed in CHARLS (37.4%). Over a median follow-up of 11.00 years (IQR, 5.00–13.00), 4,034 incident stroke events occurred (9.7%), with the highest event rate in HRS (14.8%) and the lowest in CHARLS (1.8%), a difference partially attributable to the substantially longer follow-up duration in HRS (median, 14.00 years) compared with CHARLS (median, 4.00 years) (Table 1).

**Table 1:** Baseline characteristics of study participants by cohort

Characteristics	Level	Overall (*n* = 41,717)	CHARLS (*n* = 7,118)	HRS (*n* = 14,806)	ELSA (*n* = 3,973)	SHARE (*n* = 15,820)	*p* value	SMD
Age, years		62.00 [56.00, 69.00]	59.00 [52.00, 65.00]	64.00 [59.00, 72.00]	62.00 [57.00, 69.00]	61.00 [55.00, 69.00]	<0.001	0.310
Gender	Female	23,671 (56.7)	3,815 (53.6)	8,746 (59.1)	2,238 (56.3)	8,872 (56.1)	<0.001	0.047
	Male	18,046 (43.3)	3,303 (46.7)	6,060 (40.9)	1,735 (43.7)	6,948 (43.9)	–	–
Education level	High	8,389 (20.1)	1,511 (21.2)	2,842 (19.2)	677 (17.0)	3,359 (21.2)	<0.001	0.376
	Low	16,485 (39.5)	3,852 (54.1)	3,605 (24.3)	1,312 (33.0)	7,716 (48.8)	–	–
	Medium	16,843 (40.4)	1,755 (24.7)	8,359 (56.5)	1,984 (49.9)	4,745 (30.0)	–	–
Marital status	Divorced	3,696 (8.9)	336 (4.7)	1,717 (11.6)	472 (11.9)	1,171 (7.4)	<0.001	0.241
	Married	30,824 (73.9)	6,021 (84.6)	10,007 (67.6)	2,822 (71.0)	11,974 (75.7)	–	–
	Single	1,444 (3.5)	40 (0.6)	421 (2.8)	171 (4.3)	812 (5.1)	–	–
	Widowed	5,753 (13.8)	721 (10.1)	2,661 (18.0)	508 (12.8)	1,863 (11.8)	–	–
Residence (%)	Rural	12,927 (34.2)	4,872 (68.4)	4,333 (29.3)	–	3,722 (23.5)	<0.001	0.511
	Urban	24,817 (65.8)	2,246 (31.6)	10,473 (70.7)	–	12,098 (76.5)	–	–
Smoking status	No	33,829 (81.1)	4,939 (69.4)	12,565 (84.9)	3,477 (87.5)	12,848 (81.2)	<0.001	0.201
	Yes	7,888 (18.9)	2,179 (30.6)	2,241 (15.1)	496 (12.5)	2,972(18.8)	–	–
Drinking status	No	17,216 (41.3)	4,829 (67.8)	7,620 (51.5)	310 (7.8)	4,457 (28.2)	<0.001	0.679
	Yes	24,501 (58.7)	2,289 (32.2)	7,186 (48.5)	3,663 (92.2)	11,363 (71.8)	–	–
Depression	No	31,515 (75.5)	4,459 (62.6)	11,678 (78.9)	3,235 (81.4)	12,143 (76.8)	<0.001	0.186
	Yes	10,202 (24.5)	2,659 (37.4)	3,128 (21.1)	738 (18.6)	3,677 (23.2)	–	–
Hypertension	No	27 477 (65.9)	5,378 (75.6)	8,453 (57.1)	2,594 (65.3)	11,052 (69.9)	<0.001	0.178
	Yes	14,240 (34.1)	1,740 (24.4)	6,353 (42.9)	1,379 (34.7)	4,768 (30.1)	–	–
Diabetes mellitus	No	37,829 (90.7)	6,714 (94.3)	12,947 (87.4)	3,743 (94.2)	14,425 (91.2)	<0.001	0.123
	Yes	3,888 (9.3)	404 (5.7)	1,859 (12.6)	230 (5.8)	1,395 (8.8)	–	–
Heart disease	No	36,775 (88.2)	6,317 (88.7)	12,329 (83.3)	3,833 (96.5)	14,296 (90.4)	<0.001	0.196
	Yes	4,942 (11.8)	801 (11.3)	2,477 (16.7)	140 (3.5)	1,524 (9.6)	–	–
Cancer	No	39,312 (94.2)	7,059 (99.2)	13,379 (90.4)	3,727 (93.8)	15,147 (95.7)	<0.001	0.186
	Yes	2,405 (5.8)	59 (0.8)	1,427 (9.6)	246 (6.2)	673 (4.3)	–	–
Arthritis	No	27,910 (66.9)	4,730 (66.5)	7,484 (50.5)	2,736 (68.9)	12,960 (81.9)	<0.001	0.287
	Yes	13,807 (33.1)	2,388 (33.5)	7,322 (49.5)	1,237 (31.1)	2,860 (18.1)	–	–
Chronic lung disease	No	39,409 (94.5)	6,439 (90.5)	13,991 (94.5)	3,804 (95.7)	15,175 (95.9)	<0.001	0.100
	Yes	2,308 (5.5)	679 (9.5)	815 (5.5)	169 (4.3)	645 (4.1)	–	–
Incident stroke	No	37,683 (90.3)	6,991 (98.2)	12,611 (85.2)	3,768 (94.8)	14,313 (90.5)	<0.001	0.235
	Yes	4,034 (9.7)	127 (1.8)	2,195 (14.8)	205 (5.2)	1,507 (9.5)	–	–
Follow-up time, years		11.00 [5.00, 13.00]	4.00 [4.00, 4.00]	14.00 [8.00, 20.00]	12.00 [12.00, 12.00]	11.00 [7.00, 13.00]	<0.001	1.560

### Longitudinal distributions of BMI and frailty index

**Figure 2:**
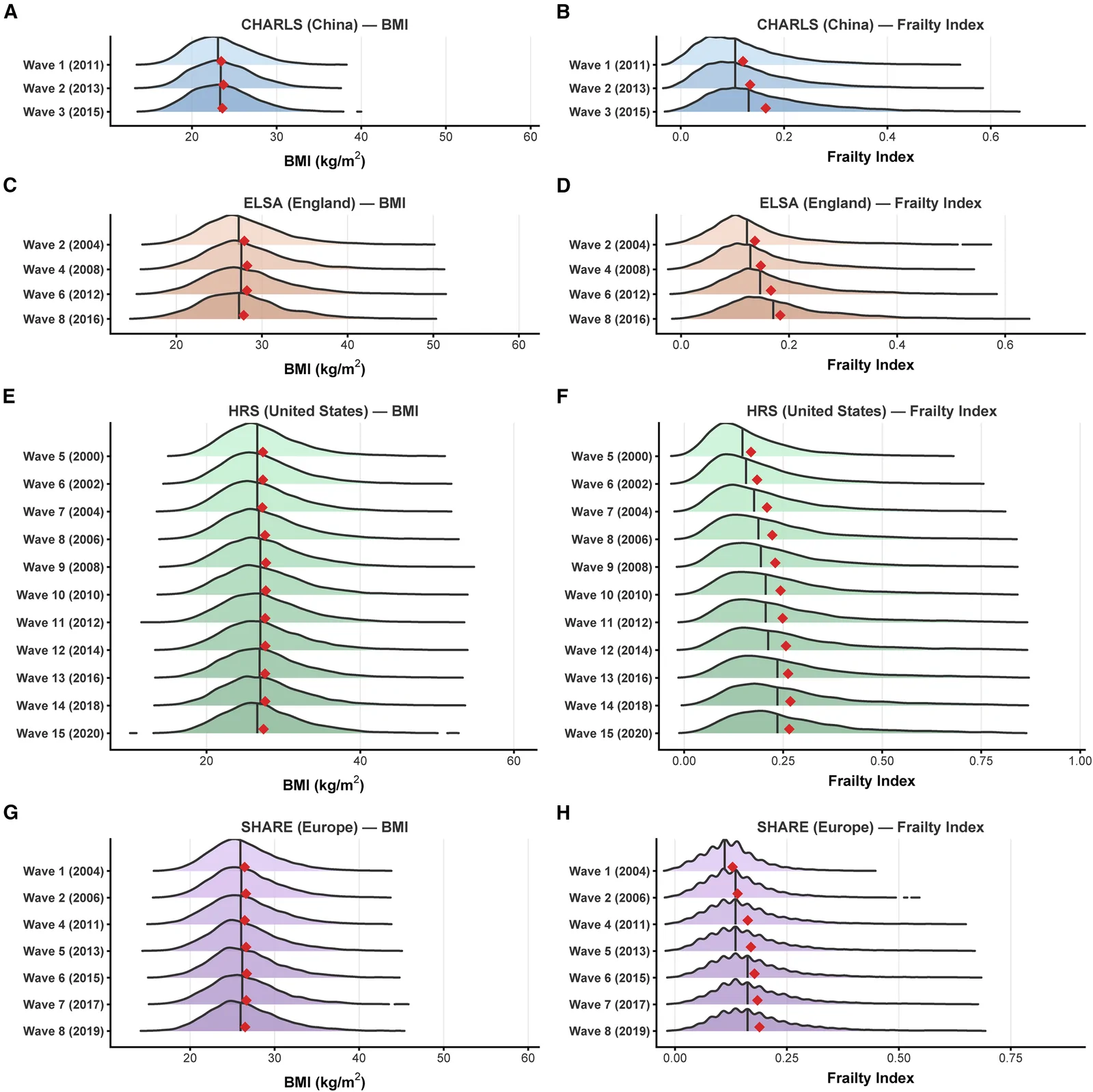
Ridge density plots of BMI and frailty index distributions across survey waves in CHARLS, ELSA, HRS, and SHARE (A) CHARLS-BMI. (B) CHARLS-frailty index. (C) ELSA-BMI. (D) ELSA-frailty index. (E) HRS-BMI. (F) HRS-frailty index. (G) SHARE-BMI. (H) SHARE-frailty index. Red diamonds indicate wave-specific means. Vertical lines within density ridges represent medians. Color gradients within each cohort represent successive survey waves.

Across all four cohorts, BMI distributions remained relatively stable over the observation period, whereas frailty index (FI) showed a consistent upward trajectory (Figure 2; Tables S1 and S2). CHARLS participants maintained the lowest mean BMI values across all waves (range, 23.45–23.72 kg/m^2^) (Figure 2A), whereas ELSA participants exhibited the highest levels, peaking at 28.27 kg/m^2^ at wave 4 before declining modestly to 27.87 kg/m^2^ at wave 8 (Figure 2C). HRS mean BMI values fluctuated narrowly between 27.26 and 27.72 kg/m^2^ over 20 years of follow-up (Figure 2E), and SHARE values ranged from 26.46 to 26.70 kg/m^2^ across seven waves (Figure 2G). In contrast, FI demonstrated progressive increases in every cohort. HRS exhibited the most pronounced absolute increase, with mean FI rising from 0.1687 at wave 5 (2000) to 0.2652 at wave 15 (2020) (Figure 2F). CHARLS experienced the steepest relative increase, with mean FI rising 36.6% from 0.1205 to 0.1646 over just four years (Figure 2B). ELSA and SHARE showed steady increases from approximately 0.13 at baseline to 0.18–0.19 at their respective final waves (Figures 2D and 2H). Across all cohorts, FI distributions were right-skewed with progressively widening interquartile ranges over successive waves, indicating increasing heterogeneity in frailty burden with aging (Table S2).

### Identification of BMI-frailty trajectory groups and stroke incidence

**Figure 3:**
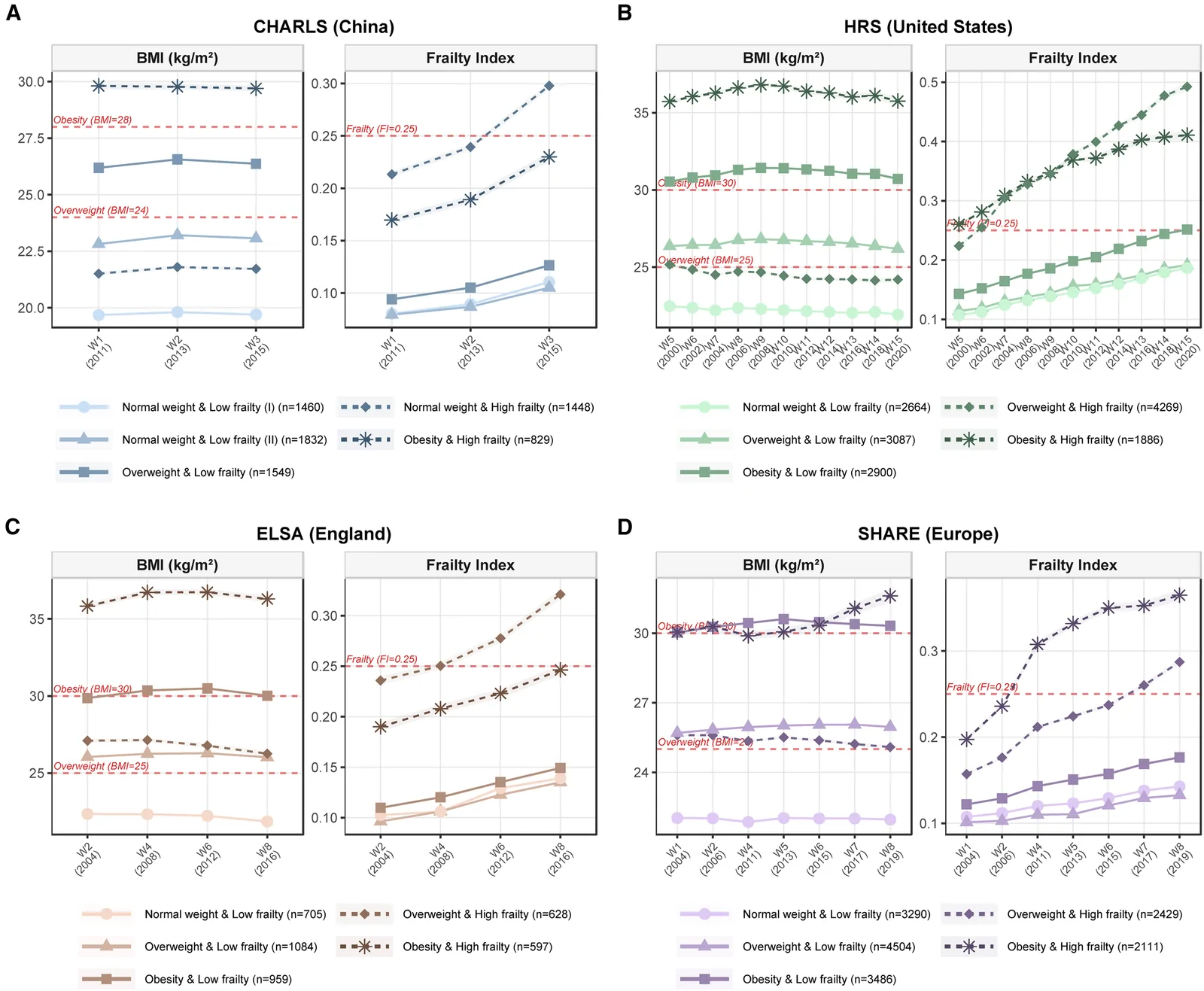
Joint BMI-frailty index trajectories identified by group-based multi-trajectory modeling (K = 5) in four cohort studies (A) CHARLS (China). (B) HRS (United States). (C) ELSA (England). (D) SHARE (Europe). Left images display BMI trajectories. Right images display frailty index trajectories. Points represent group-specific means at each wave, with shaded bands indicating standard errors. Dashed horizontal red lines denote clinical thresholds for overweight, obesity, and frailty (FI = 0.25). Solid lines indicate low-frailty groups. Dashed lines indicate high-frailty groups.

Building on these longitudinal trends, group-based multi-trajectory modeling (GBMTM) was applied to identify joint-trajectory subgroups. Model selection criteria including Akaike information criterion (AIC), Bayesian information criterion (BIC), and Gap statistic, consistently supported K = 5 as the optimal number of groups across all four cohorts (Figure S1). Five distinct trajectory groups were identified in each cohort, characterized by combinations of weight status (normal weight, overweight, or obesity) and frailty level (low or high) (Figure 3). In HRS (Figure 3B), the five groups were normal weight and low frailty (*n* = 2,664; baseline BMI, 22.47 kg/m^2^; and FI, 0.1069), overweight and low frailty (*n* = 3,087), obesity and low frailty (*n* = 2,900), overweight and high frailty (*n* = 4,269), and obesity and high frailty (*n* = 1,886; baseline BMI, 35.73 kg/m^2^; and FI, 0.2602 rising to 0.4107 over 20 years). Similar patterns emerged in CHARLS (Figure 3A), ELSA (Figure 3C), and SHARE (Figure 3D), with high-frailty groups consistently exhibiting steeper FI trajectories, whereas BMI remained relatively stable within each weight category. In CHARLS, two distinct normal-weight and low-frailty subgroups were identified, differing slightly in baseline characteristics.

Cumulative incidence of stroke differed significantly across trajectory groups in all cohorts (log rank *p* < 0.001 for all; Figure S2). High-frailty groups consistently showed the highest stroke event rates. In HRS, the overweight and high-frailty group had the highest rate (25.35%), compared with 7.58% in the normal-weight and low-frailty group (Figure S2B). In SHARE, the obesity and high-frailty group reached 24.59% versus 4.02% in the overweight and low-frailty group (Figure S2D). ELSA showed 12.10% for the overweight and high-frailty group versus 2.86% for overweight and low frailty (Figure S2C), and CHARLS exhibited the lowest overall rates, with the normal-weight and high-frailty group at 3.94% versus 0.62% for the normal-weight and low-frailty (I) group (Figure S2A).

### Association between BMI-frailty trajectory groups and incident stroke

**Figure 4:**
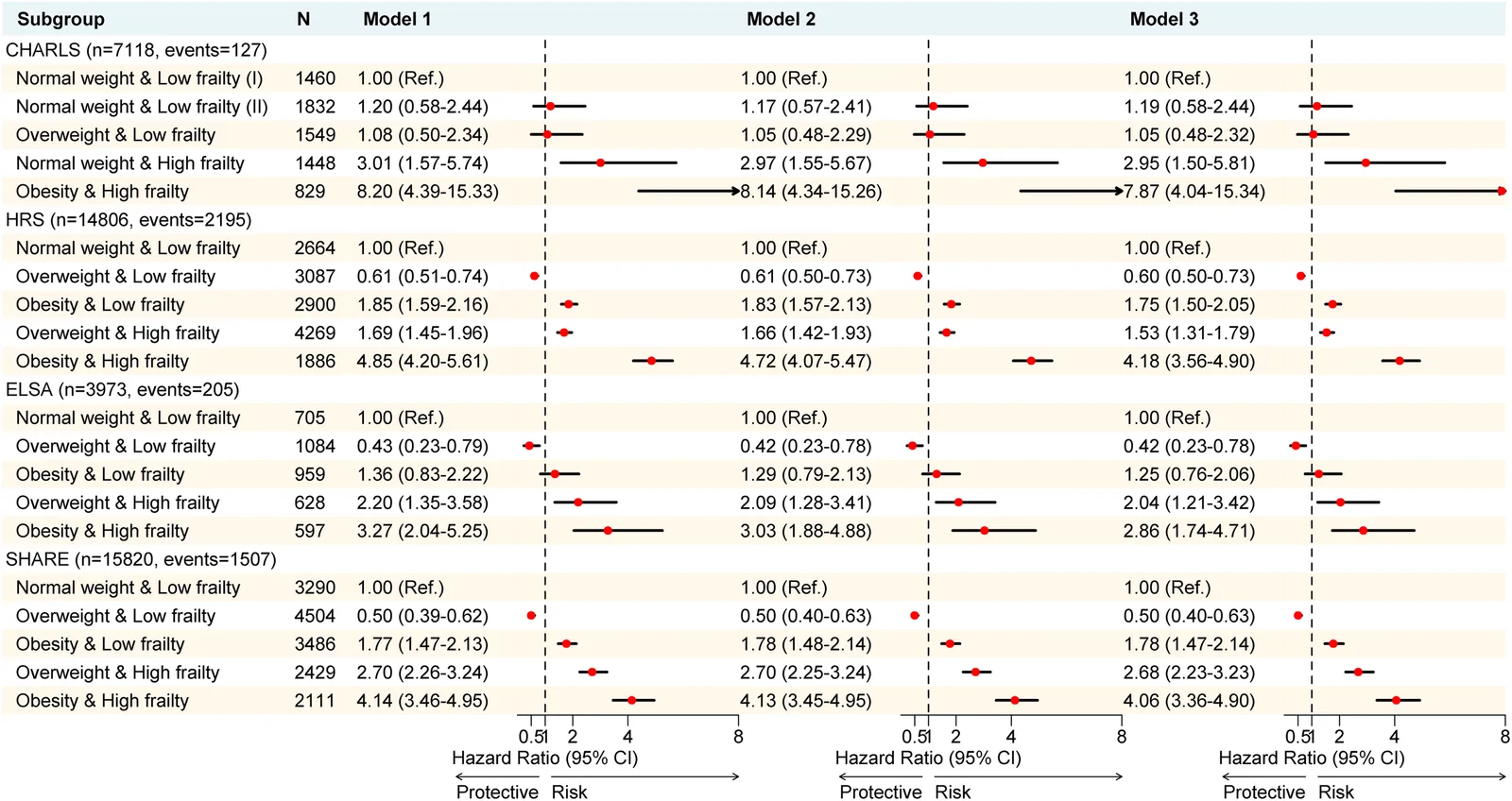
Forest plot of hazard ratios for incident stroke by BMI-frailty trajectory group across four cohort studies Three progressively adjusted Cox proportional hazards models are presented: model 1 (age and sex); model 2 (model 1 + education, marital status, and residence); model 3 (model 2 + smoking, drinking, depression, hypertension, diabetes, heart disease, cancer, arthritis, and chronic lung disease). The “normal-weight and low-frailty” group serves as the reference (HR = 1.00). Squares represent point estimates, and horizontal lines represent 95% confidence intervals.

Cox proportional hazards models with progressive covariate adjustment quantified these associations (Figure 4). In the fully adjusted model (model 3), the obesity and high-frailty group demonstrated the highest stroke risk across all cohorts: CHARLS (hazard ratio [HR] = 7.87, 95% confidence interval [CI], 4.04–15.34, *p* < 0.001), HRS (HR = 4.18, 95% CI, 3.56–4.90, *p* < 0.001), ELSA (HR = 2.86, 95% CI, 1.74–4.71, *p* < 0.001), and SHARE (HR = 4.06, 95% CI, 3.36–4.90, *p* < 0.001). The overweight and high-frailty group was also significantly associated with elevated risk in HRS (HR = 1.53, 95% CI, 1.31–1.79), ELSA (HR = 2.04, 95% CI, 1.21–3.42), and SHARE (HR = 2.68, 95% CI, 2.23–3.23). In CHARLS, the normal-weight and high-frailty group showed a nearly 3-fold increased risk (HR = 2.95, 95% CI, 1.50–5.81, *p* = 0.002). Conversely, the overweight and low-frailty group exhibited a protective association in HRS (HR = 0.60, 95% CI, 0.50–0.73), ELSA (HR = 0.42, 95% CI, 0.23–0.78), and SHARE (HR = 0.50, 95% CI, 0.40–0.63; all *p* < 0.001). HRs remained stable across the three progressively adjusted models within each cohort, indicating robustness to confounding adjustment.

### Subgroup and sensitivity analyses

The robustness of the primary findings was evaluated through extensive subgroup and sensitivity analyses. Stratification by age, sex, education level, marital status, residence, smoking, drinking, and major comorbidities yielded consistent results, with the obesity and high-frailty group maintaining the highest stroke risk across nearly all strata in each cohort; most interaction *p* values were non-significant (Table S3). Five sensitivity analyses further confirmed the stability of the main findings (Table S4). When World War I replaced BMI as the adiposity measure, the obesity and high-frailty group remained strongly associated with stroke in all cohorts (e.g., CHARLS: HR = 8.14, 95% CI, 4.15–15.97; HRS: HR = 4.35, 95% CI, 3.70–5.12; SHARE: HR = 4.22, 95% CI, 3.49–5.10; all *p* < 0.001). Substituting the Fried frailty phenotype for the FI produced slightly attenuated but directionally consistent estimates (e.g., HRS: HR = 3.85, 95% CI, 3.27–4.54 and SHARE: HR = 3.72, 95% CI, 3.07–4.51). Fine-Gray competing risk models accounting for mortality yielded modestly reduced subdistribution HRs (sHRs), whereas all associations remained statistically significant (e.g., CHARLS: sHR = 6.89, 95% CI, 3.52–13.48 and HRS: sHR = 3.76, 95% CI, 3.20–4.42). Excluding underweight participants (BMI <18.5 kg/m^2^) and randomly removing 20% of the sample produced virtually identical results to the primary analysis.

## Discussion

### Summary of principal findings

This multi-cohort study, encompassing 41,717 participants from four nationally representative aging surveys across 18 countries, identified five distinct joint BMI-frailty trajectory groups using group-based multi-trajectory modeling and examined their associations with incident stroke. The principal finding was that the obesity and high-frailty trajectory group consistently demonstrated the highest stroke risk across all four cohorts, with fully adjusted HRs ranging from 2.86 in ELSA to 7.87 in CHARLS, compared with the normal-weight and low-frailty reference group. A secondary but equally important finding was that high frailty, rather than elevated BMI alone, appeared to be the dominant driver of stroke risk: within the same BMI category, high-frailty groups invariably exhibited substantially higher stroke incidence than their low-frailty counterparts. Notably, the overweight and low-frailty group showed a consistently protective association in HRS, ELSA, and SHARE, with HRs of 0.42–0.60. These associations remained robust across three progressively adjusted models, extensive subgroup analyses, and five sensitivity analyses including alternative adiposity measures, competing risk models, and random sample exclusion.

### The synergistic effect of obesity and frailty on stroke risk

Our finding that the co-occurrence of obesity and high frailty confers the greatest stroke risk extends prior evidence that has examined these factors in isolation. A meta-analysis of 14 studies reported a pooled pre-stroke frailty prevalence of 24.6% and demonstrated that frailty was associated with longer-term mortality with an odds ratio of 3.75.^5^ Similarly, an observational study of over 253,000 participants from the All of Us research program demonstrated a striking dose-response relationship between hospital frailty risk scores and stroke risk, with HRs escalating from 4.9 for low frailty to 42.8 for high frailty compared with non-frail individuals.^6^ A recent dose-response meta-analysis further confirmed that each one-point increase in frailty score was associated with a 6.4% higher risk of death among stroke survivors.^4^ Our study builds upon these findings by demonstrating that frailty status modifies the obesity-stroke association in a trajectory-dependent manner, an insight not captured by cross-sectional or single-exposure analyses.

The observation that high-frailty groups within the same BMI category consistently showed elevated stroke risk aligns with emerging evidence from a study of over three million US Veterans, which demonstrated that both the presence and severity of frailty were tightly correlated with cardiovascular mortality and stroke risk independent of underlying cardiovascular disease.^12^ Furthermore, a World Stroke Organization scientific statement has emphasized that frailty has important disease- and treatment-modifying effects across the entire stroke pathway, advocating for routine frailty assessment in stroke risk stratification.^13^ Our trajectory-based approach provides a longitudinal dimension to these cross-sectional observations, revealing that sustained co-exposure to obesity and progressive frailty amplifies stroke risk beyond what either condition alone would predict.

### The overweight-low frailty paradox and metabolic heterogeneity

A particularly intriguing finding was the protective association observed for the overweight and low-frailty trajectory group across three Western cohorts. This observation resonates with the well-documented obesity paradox in stroke, wherein an umbrella review of seven systematic reviews encompassing over one million participants concluded that excess body weight was associated with reduced post-stroke mortality compared with normal weight.^3^^,^^14^ Our findings extend this paradox from post-stroke outcomes to stroke incidence, suggesting that moderate adiposity coupled with preserved physiological resilience may confer a protective phenotype.

This interpretation is further supported by evidence on the metabolic heterogeneity of obesity. A study using CHARLS and ELSA cohorts demonstrated that metabolically unhealthy overweight or obesity accelerated frailty progression, whereas metabolically healthy overweight or obesity did not differ from metabolically healthy normal weight.^8^ A rural Chinese cohort study similarly showed that metabolically unhealthy obesity carried a 3.22-fold higher risk of ischemic stroke compared with metabolically healthy normal weight.^15^ These findings collectively suggest that it is the metabolic milieu accompanying obesity, rather than adiposity per se, that drives both frailty progression and stroke risk. Our overweight and low-frailty group likely represents a metabolically healthier phenotype with preserved muscle mass and functional capacity, consistent with the concept that body composition quality matters more than BMI alone.^16^

### Frailty trajectories, brain vulnerability, and stroke mechanisms

The progressive increase in frailty index observed across all cohorts, coupled with the steeper FI trajectories in high-frailty groups, aligns with a systematic review demonstrating that frailty tends to worsen over time and that baseline frailty accelerates further progression.^11^ A recent study using the same four cohorts as our analysis showed that frailty accelerates significantly both before and after incident stroke, suggesting a bidirectional relationship.^10^ This is further corroborated by Mendelian randomization evidence indicating that genetically determined frailty is independently associated with both stroke risk and worse functional outcome after ischemic stroke, supporting a potentially causal role.^17^

The mechanisms linking the obesity-frailty phenotype to stroke likely involve chronic systemic inflammation, endothelial dysfunction, and accelerated vascular aging. A meta-analysis demonstrated that sarcopenic obesity—a condition sharing pathophysiological features with our obesity and high-frailty trajectory—was associated with a 3.76-fold higher risk of frailty compared with robust individuals.^18^ Recent neuroimaging evidence has shown that brain frailty markers, including white matter disease and cortical atrophy, mediate up to 85% of the association between age and post-stroke outcomes, suggesting that systemic frailty may translate into cerebrovascular vulnerability through cumulative brain injury.^19^ Post hoc analyses of randomized trials have further confirmed that brain frailty independently predicts worse functional recovery after both thrombolysis and thrombectomy.^20^^,^^21^

### Clinical implications and future directions

This study has several strengths. First, the use of four internationally harmonized longitudinal cohorts spanning 18 countries enhances the generalizability and external validity of our findings. Second, group-based multi-trajectory modeling simultaneously captured the co-evolution of BMI and frailty, providing a more nuanced characterization than traditional single-variable approaches. Third, the consistency of findings across cohorts with markedly different population characteristics, BMI distributions, and follow-up durations strengthens the evidence for a genuine biological relationship. Fourth, the robustness of results across five sensitivity analyses, including alternative adiposity and frailty measures and Fine-Gray competing risk models, mitigates concerns about measurement artifact or confounding.

This study provides the first multi-cohort longitudinal evidence that joint BMI-frailty trajectories, rather than either measure in isolation, are powerful predictors of incident stroke in aging populations. The finding that frailty status fundamentally modifies the obesity-stroke relationship has direct clinical implications: routine frailty assessment should be integrated into stroke risk stratification alongside traditional cardiovascular risk factors, as advocated by recent international consensus statements.^9^^,^^13^ The protective association observed for the overweight and low-frailty group suggests that weight management strategies in older adults should prioritize preservation of functional capacity and metabolic health over BMI reduction alone. A multicenter cohort study of 667 stroke patients demonstrated that baseline frailty independently predicted non-elective readmission and major adverse cerebrovascular events, reinforcing the clinical utility of frailty screening in stroke populations.^22^ Future research should employ mediation analyses to disentangle the relative contributions of metabolic dysfunction, sarcopenia, and chronic inflammation to the obesity-frailty-stroke pathway. Randomized trials evaluating whether targeted interventions addressing both obesity and frailty—such as combined exercise and nutritional programs—can reduce stroke incidence in high-risk trajectory groups are warranted.

In this multi-cohort longitudinal study of 41,717 participants from CHARLS, HRS, ELSA, and SHARE, group-based multi-trajectory modeling identified five distinct joint BMI-frailty trajectory groups, among which the obesity and high-frailty group demonstrated the highest and the overweight and low-frailty group the lowest risk of incident stroke across all four cohorts, independent of demographic, behavioral, and clinical confounders. High-frailty status was consistently the dominant risk modifier within each BMI category, whereas moderate overweight coupled with preserved functional resilience was associated with a reduced stroke risk. These findings indicate that joint BMI-frailty trajectory assessment provides superior stroke risk stratification compared with either measure alone, supporting the integration of longitudinal frailty monitoring into preventive stroke care for aging populations.

### Limitations of the study

Several limitations warrant consideration. First, stroke events were ascertained through self-report of physician diagnosis, which may introduce misclassification and underestimate true incidence. Furthermore, these survey-based cohorts did not capture stroke subtypes (ischemic vs. hemorrhagic), clinical severity scores (e.g., National Institutes of Health Stroke Scale [NIHSS]), or neuroimaging findings (e.g., MRI), which limits the ability to examine differential associations across stroke subtypes and may reduce the interpretability of the findings. Second, frailty indices were constructed using available survey items that varied across cohorts, potentially limiting strict cross-cohort comparability despite harmonization efforts. Third, BMI does not capture body composition nuances such as visceral adiposity or sarcopenia, which may differentially influence stroke risk.^16^ Fourth, CHARLS had only three waves of data and a short follow-up period, limiting the precision of trajectory estimation and yielding lower stroke event rates. Fifth, residual confounding by unmeasured factors such as dietary patterns, physical activity intensity, and genetic susceptibility cannot be excluded.^23^ Finally, the observational design precludes definitive causal inference, although the consistency with Mendelian randomization evidence supports a plausible causal pathway.^6^^,^^17^

## Resource availability

### Lead contact

Requests for further information and resources should be directed to and will be fulfilled by the lead contact, Hong Liu (xinyuhong80025375@126.com).

### Materials availability

This study did not generate new unique reagents.

### Data and code availability

• This paper analyzes existing, publicly available data. CHARLS data are accessible at http://charls.pku.edu.cn/. HRS data are accessible at https://hrs.isr.umich.edu/. ELSA data are accessible at https://www.elsa-project.ac.uk/. SHARE data are accessible at http://www.share-project.org/. Access is subject to each study’s data use agreement.
• All original code has been deposited at GitHub and is publicly available at https://github.com/Paperaccepted1/Iscience.
• Any additional information required to reanalyze the data reported in this paper is available from the lead contact upon request.

## Acknowledgments

This research did not receive any specific grant from funding agencies in the public, commercial, or not-for-profit sectors.

## Author contributions

Conceptualization, Z.-J.W. and H.L.; methodology, Z.-J.W. and Q.-H.H.; software, Z.-J.W.; formal analysis, Z.-J.W.; investigation, Z.-J.W., Q.-H.H., M.-F.H., and Q.-X.L.; data curation, Z.-J.W. and M.-F.H.; writing – original draft, Z.-J.W.; writing – review and editing, H.L. and J.-R.Z.; visualization, Z.-J.W. and Q.-X.L.; supervision, H.L.; project administration, H.L.

## Declaration of interests

The authors declare no competing interests.

## Declaration of generative AI and AI-assisted technologies in the writing process

During the preparation of this work, the author(s) used ChatGPT/Claude in order to assist with language polishing and editing. After using this tool, the author(s) reviewed and edited the content as needed and take(s) full responsibility for the content of the publication.

## STAR★Methods

### Key resources table

**Table:** 

REAGENT or RESOURCE	SOURCE	IDENTIFIER
**Deposited data**		

China Health and Retirement Longitudinal Study (CHARLS)	Peking University	http://charls.pku.edu.cn/
Health and Retirement Study (HRS)	University of Michigan	https://hrs.isr.umich.edu/
English Longitudinal Study of Ageing (ELSA)	University College London	https://www.elsa-project.ac.uk/
Survey of Health, Ageing and Retirement in Europe (SHARE)	SHARE-ERIC	http://www.share-project.org/

**Software and algorithms**		

R (version 4.3)	R Foundation for Statistical Computing	https://www.r-project.org/
traj package (for group-based multi-trajectory modeling)	CRAN	https://cran.r-project.org/web/packages/traj/
survival package (for Cox proportional hazards models)	CRAN	https://cran.r-project.org/web/packages/survival/
cmprsk package (for Fine–Gray competing risk models)	CRAN	https://cran.r-project.org/web/packages/cmprsk/
Original analysis code	This paper	https://github.com/Paperaccepted1/Iscience

### Experimental model and study participant details

#### Study design and data sources

This harmonised longitudinal analysis used data from four nationally representative aging cohorts: the China Health and Retirement Longitudinal Study (CHARLS, 2011–2015, 4 waves),^24^^,^^25^ the Health and Retirement Study (HRS, United States, 2000–2020, 11 waves),^26^ the English Longitudinal Study of Ageing (ELSA, 2004–2016, 4 waves),^27^ and the Survey of Health, Ageing and Retirement in Europe (SHARE, 2004–2019, 7 waves).^28^ Details of each cohort's design have been published previously. CHARLS was approved by the Biomedical Ethics Review Committee of Peking University (IRB00001052-11015). HRS was approved by the University of Michigan Institutional Review Board (HUM00061128). ELSA was approved by the South Central – Berkshire Research Ethics Committee (17/SC/0588). SHARE was approved by the Ethics Committee of the University of Munich (142/16_S). All participants provided informed consent.

#### Study population

All four cohorts adopted a consistent stroke definition based on self-reported physician diagnosis. The age inclusion criterion was unified at ≥45 years. Each cohort employed its own nationally representative sampling strategy, and the number of survey waves and follow-up durations varied across cohorts. Cohort-specific analyses were conducted rather than pooled analyses to preserve sample integrity and account for cross-cohort heterogeneity in sampling design and follow-up duration. Figure 1 presents the participant selection flowchart. Participants were excluded if they: (1) were younger than 45 years of age; (2) had missing body mass index (BMI) or frailty data at baseline; or (3) had been diagnosed with stroke prior to or at baseline. After applying these exclusion criteria, the final analytic sample comprised 7,118 participants from CHARLS (baseline Wave 1, with 4 waves of data), 14,806 from HRS (baseline Wave 5, with 15 waves), 3,973 from ELSA (baseline Wave 2, with 8 waves), and 15,820 from SHARE (baseline Wave 1, with 8 waves), yielding a total of 41,717 participants. Participants were not randomized to experimental groups. Group assignment was determined empirically through group-based multi-trajectory modeling (GBMTM), which identified five latent trajectory subgroups within each cohort based on joint longitudinal patterns of BMI and frailty index. Group membership was assigned according to each participant's highest posterior probability of belonging to a given trajectory group.

### Method details

#### Body mass index

BMI (kg/m^2^) was derived from measured weight and height at each wave. Obesity was defined as BMI ≥28 kg/m^2^ for CHARLS (Chinese standards) and ≥30 kg/m^2^ for the remaining cohorts (WHO criteria).

#### Frailty index

A deficit-accumulation frailty index (FI) was constructed for each cohort following established methodology.^29^ The FI included 27 items for HRS, CHARLS, and ELSA, and 26 items for SHARE, encompassing chronic diseases (excluding heart disease and stroke), self-reported health, functional disabilities, and cognition. All items except cognition were dichotomized (1 = deficit, 0 = no deficit); cognition was treated as a continuous variable ranging from 0 to 1, with higher scores indicating poorer cognitive function. The FI was calculated as the ratio of deficits present to total deficits assessed, yielding a score from 0 to 1. Frailty was defined as FI ≥ 0.25.

#### Incident stroke

The primary outcome was self-reported physician-diagnosed incident stroke occurring after baseline. Follow-up was calculated from the baseline wave to the wave of first stroke report or last available assessment.

#### Covariates

Baseline covariates included age, sex, education, marital status, residence (urban/rural; unavailable for ELSA), smoking and drinking status, and self-reported physician-diagnosed comorbidities (depression, hypertension, diabetes, heart disease, cancer, arthritis, and chronic lung disease).

### Quantification and statistical analysis

#### Group-based multi-trajectory modelling

Group-based multi-trajectory modelling (GBMTM) was used to identify latent subgroups sharing similar joint BMI–FI trajectories within each cohort. BMI and FI were standardised to z-scores prior to modelling, and quadratic polynomial functions were specified. The optimal number of groups (K=2–6) was determined by Bayesian Information Criterion, Akaike Information Criterion, and Gap statistic, supplemented by clinical interpretability. Average posterior probabilities exceeding 0.80 indicated acceptable classification accuracy.

#### Survival analysis

Kaplan–Meier curves estimated cumulative stroke incidence by trajectory group, with between-group differences tested by log-rank test. Cox proportional hazards models estimated hazard ratios (HRs) with 95% confidence intervals (CIs) using three progressively adjusted models: Model 1 (age, sex); Model 2 (plus education, marital status, residence); Model 3 (plus smoking, drinking, depression, hypertension, diabetes, heart disease, cancer, arthritis, chronic lung disease). The Normal weight & Low frailty group served as the reference.

#### Subgroup and sensitivity analyses

Subgroup analyses stratified by age (<65 vs ≥65 years), sex, and major comorbidities were conducted, with interaction effects assessed by likelihood ratio tests. Five sensitivity analyses evaluated robustness: (1) replacing BMI with weight-adjusted waist index; (2) substituting Fried frailty phenotype for FI; (3) excluding underweight participants (BMI <18.5 kg/m^2^); (4) Fine–Gray competing-risk models accounting for mortality; and (5) random 20% sample removal with re-estimation. All analyses were performed in R (version 4.3), with two-sided *p* <0.05 considered significant. Sex was included as a covariate in all adjusted models. Subgroup analyses stratified by sex were conducted to evaluate potential sex-based differences in the associations between BMI–frailty trajectory groups and incident stroke, with interaction effects assessed by likelihood ratio tests (Table S3). No significant effect modification by sex was observed in most cohorts and trajectory groups.

## Supplemental information

Document S1. Figures S1 and S2

Table S1. Descriptive statistics of BMI by survey wave and cohort

Table S2. Descriptive statistics of frailty index by survey wave and cohort

Table S3. Results of subgroup analyses: hazard ratios for incident stroke by BMI-frailty trajectory group and interaction *p* values across four cohort studies

Table S4. Sensitivity analysis results: hazard ratios for incident stroke under alternative analytic specifications
