# Supplementary material for: Co-evolving adiposity and frailty trajectories shape stroke risk in middle-aged and older adults
Source: iScience. 2026 Aug 19;29(9):116954. doi: 10.1016/j.isci.2026.116954 (PMC13520624; doi:10.1016/j.isci.2026.116954)
Supplement: Supplementary file 1 [file mmc1.pdf]

iScience, Volume *29*

Supplemental information

Co-evolving adiposity and frailty trajectories

shape stroke risk in middle-aged and older adults

Zheng-Jun Wu, Qian-Hui Hu, Ming-Fang He, Qiu-Xia Liang, Jin-Rong Zhang, and Hong Liu

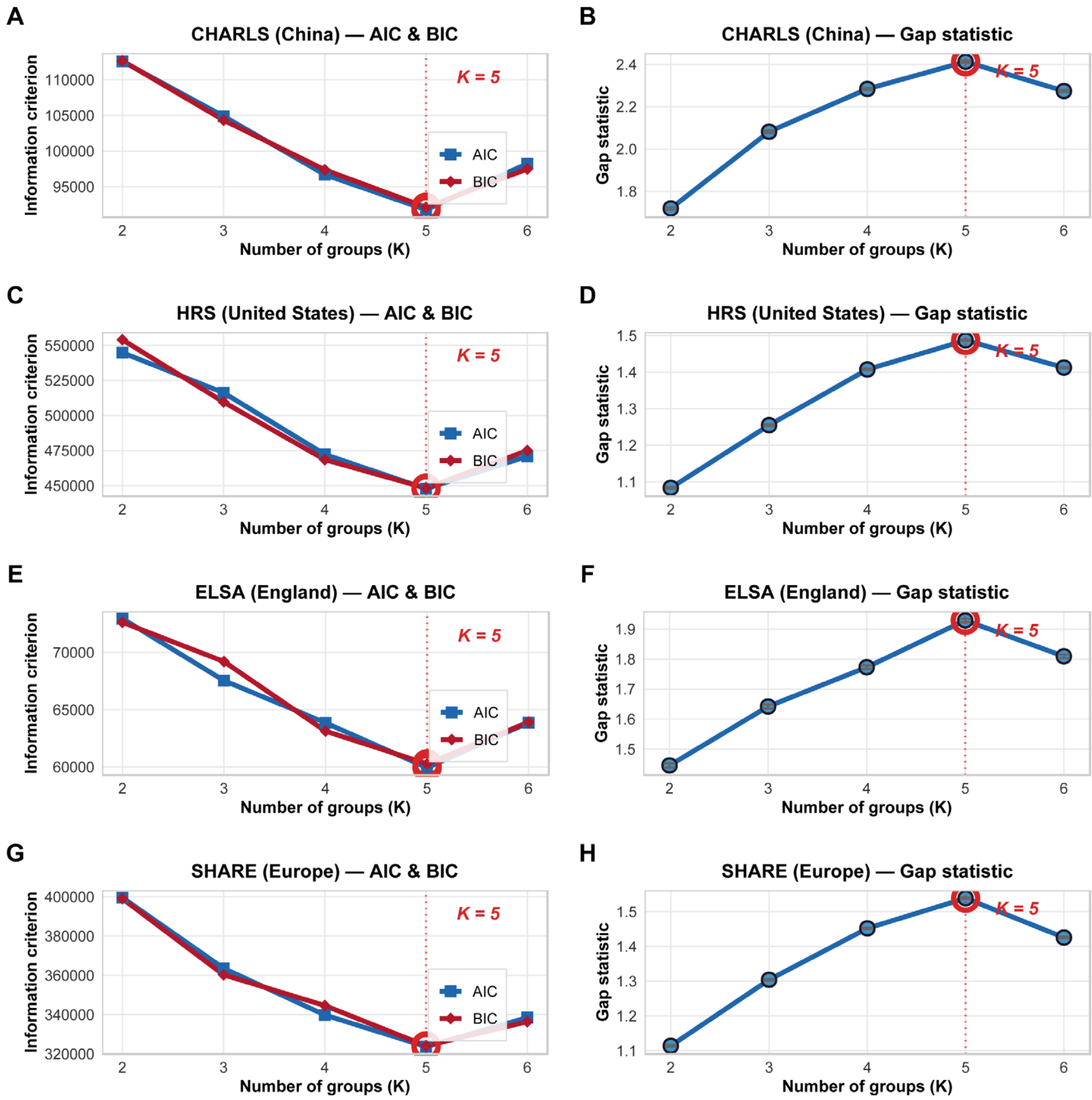

Supplementary Figure S1. Model Selection Criteria for Determining the Optimal Number of Trajectory Groups (K = 2–6) in Four Cohort Studies. (A, B) CHARLS; (C, D) HRS; (E, F) ELSA; (G, H) SHARE. Left panels show AIC and BIC across K values; right panels show Gap statistic with standard error bars. Red circles and vertical dotted lines indicate the selected optimal K = 5.

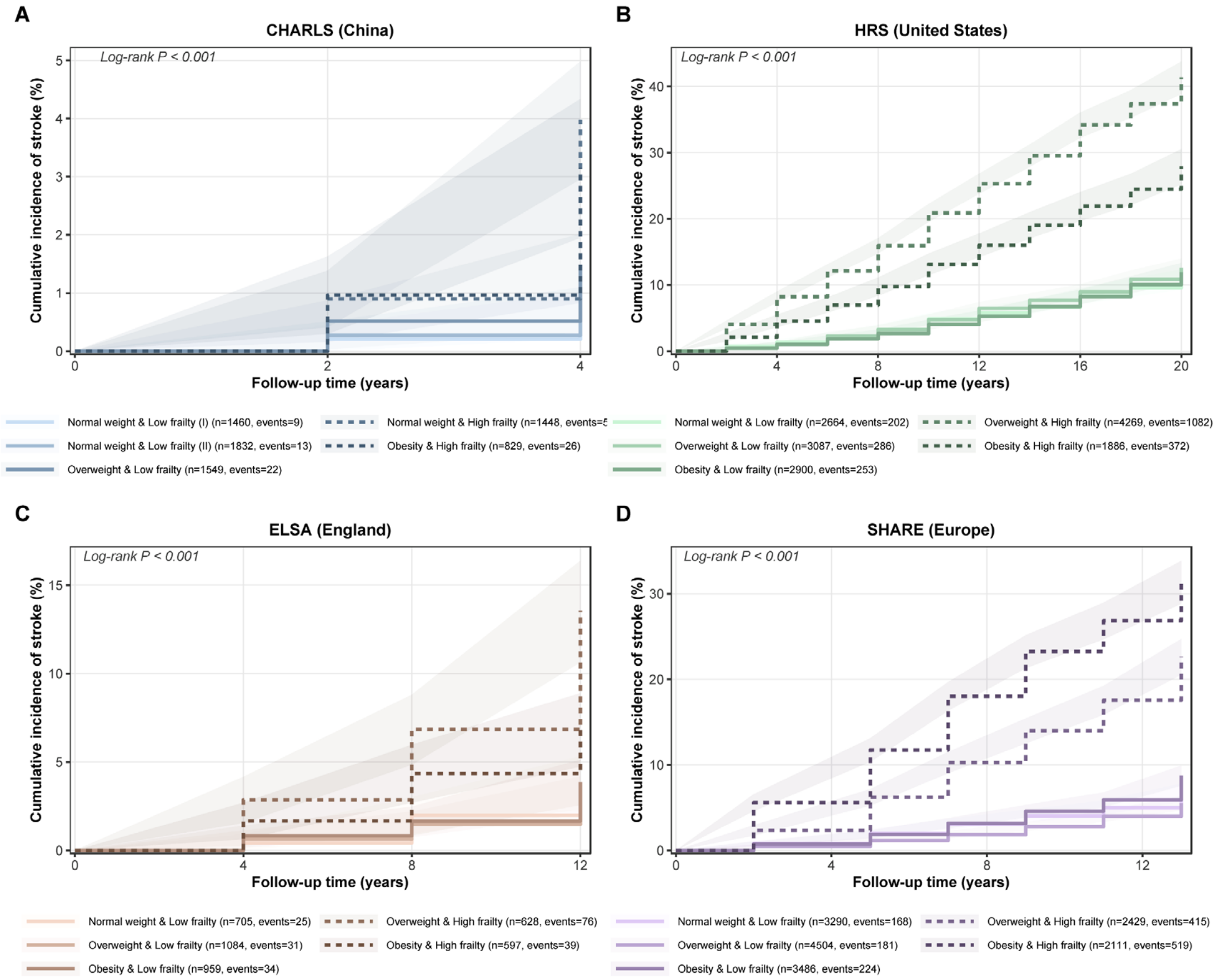

Supplementary Figure S2. Cumulative Incidence Curves of Stroke by BMI–Frailty Trajectory Group in Four Cohort Studies. (A) CHARLS (China); (B) HRS (United States); (C) ELSA (England); (D) SHARE (Europe). Cumulative incidence (%) is plotted as 1 minus the Kaplan-Meier survival estimate. Shaded bands represent 95% confidence intervals. Log-rank test P values are shown.
